# Increased calcium channel in the lamina propria of aging rat

**DOI:** 10.18632/aging.102284

**Published:** 2019-10-31

**Authors:** Ji Min Kim, Hyoung-Sam Heo, Sung-Chan Shin, Hyun-Keun Kwon, Jin-Choon Lee, Eui-Suk Sung, Hyung-Sik Kim, Gi Cheol Park, Byung-Joo Lee

**Affiliations:** 1 Biomedical Research Institute, Pusan National University Hospital, Busan, Republic of Korea; 2 Division of Bio-Medical Informatics, Center for Genome Science, Korea National Institute of Health, Korea Centers for Disease Control and Prevention, Cheongju-si, Republic of Korea; 3 Department of Otorhinolaryngology-Head and Neck Surgery, Pusan National University School of Medicine, Pusan National University, Busan, Republic of Korea; 4, Department of Otorhinolaryngology-Head and Neck Surgery, Pusan National University School of Medicine and Biomedical Research Institute, Pusan National University Yangsan Hospital, Yangsan, Republic of Korea; 5 Department of Life Science in Dentistry, school of Dentistry, Pusan National University, Yangsan, Republic of Korea; 6 Institute for Translational Dental Science, Pusan National University, Yangsan, Republic of Korea; 7 Department of Otolaryngology-Head and Neck Surgery, Samsung Changwon Hospital, Sungkyunkwan University School of Medicine, Changwon, Republic of Korea

**Keywords:** aging, vocal fold, calcium channel, extracellular matrix

## Abstract

The alterations of the extracellular matrix (ECM) in lamina propria of the vocal folds are important changes that are associated with decreased vibrations and increased stiffness in aging vocal fold. The aim of this study was to investigate the differences in gene expression of lamina propria using next generation sequencing (NGS) in young and aging rats and to identify genes that affect aging-related ECM changes for developing novel therapeutic target molecule. Among the 40 genes suggested in the NGS analysis, voltage-gated calcium channels (VGCC) subunit alpha1 S (CACNA1S), VGCC auxiliary subunit beta 1 (CACNB1), and VGCC auxiliary subunit gamma 1 (CACNG1) were increased in the lamina propria of the old rats compared to the young rats. The synthesis of collagen I and III in hVFFs decreased after si-CACNA1S and verapamil treatment. The expression and activity of matrix metalloproteinases (MMP)-1 and -8 were increased in hVFFs after the treatment of verapamil. However, there was no change in the expression of MMP-2 and -9. These results suggest that some calcium channels may be related with the alteration of aging-related ECM in vocal folds. Calcium channel has promising potential as a novel therapeutic target for the remodeling ECM of aging lamina propria.

## INTRODUCTION

The voice changes with age. The change of voice due to aging after the sixth decade of the life is called presbylaryx. Aging voices are characterized by hoarse, weak, strained, low pitch, or breathy [1–2]. Laryngostroboscopic findings of aging larynx may reveal the vocal fold bowing, glottis gap, asymmetry of vibration and decreased mucosal wave [3]. Elderly person who are aware of the increased vocal roughness and voice deterioration report a tendency to avoid social situation [4]. The impairment of communication reduces the quality of life by avoiding the social activities of the elderly. With the increase in the global elderly population, there is a growing interest in aging related voice changes.

The change of aging voice is related with systemic body condition, such as systemic medical condition, decreased pulmonary function, and neurologic disorders [2, 5]. Also, the change of histo-morphology of laryngeal muscle and vocal fold lamina propria play a crucial role in the development of aging voice. Among the histo-morphologic changes seen with aging vocal fold, the changes of the viscoelasticity of the vocal folds are important changes that are associated with decreased vibrations and increased stiffness of the vocal folds. The alterations to the extracellular matrix (ECM) of the lamina propria are the most significant findings and result in reduced viscoelasticity. In the aging lamina propria, the increase of collagen I and III and the decrease of hyaluronic acid and elastin are important changes [6–9].

The current management of aging voices is a speech therapy that improves lung function and reduces laryngeal tension [10]. Surgical treatment has been reported for the injection laryngoplasty or medialization thyroplasty [11, 12]. Regenerative medicine and tissue engineering are novel strategies for the treatment of aging vocal folds. It has been reported as the encouraging results in several studies using growth factors, such as basic fibroblast growth factor and hepatocyte growth factor [13, 14]. Although recent tissue engineering and growth factors studies have been reported to be effective in the treatment of aging vocal folds, further basic and clinical investigations are required to overcome age-related voice change because the mechanism of aging ECM changes has not yet been fully understood. The studies for the molecular mechanism of the alterations of aging-related ECM in vocal fold lamina propria may be better understood for the basic mechanism of presbylarynx and may be applied to develop the novel therapeutic target molecules for aging voice disorder. The aim of this study was to investigate the differences in genes expression of lamina propria using next generation sequencing (NGS) in young and aging rats and to identify genes that affect aging-related ECM changes for developing novel therapeutic target molecule. The studies for the molecular mechanism of the alterations of aging-related ECM in lamina propria may be better understood for the basic mechanism of presbylarynx and may be applied to develop the novel therapeutic target molecules for aging voice disorder.

## RESULTS

### Statistical analysis of gene expression changes by NGS study

The changes in age-induced gene expression over 22 months old rats compared to 6 months old rats were first evaluated at each of the group points (Table 2A). And then, we obtained corresponding annotation results as each DEG mapped into the well annotated genes (Table 2B). Detailed information for Table 1A and Table 1B shown in Supplementary data for Table 1 and Supplementary data for Table 2. At this point, we determined vocal fold aging related genes that are common between each sample comparison (Y1-4 vs O1-4, Y1-4 vs O5-8, Y5-8 vs O1-4, Y5-8 vs O5-8, and Y1-4, Y5-8 vs O1-4, O5-8). We identified 40 and 79 differentially expressed genes by aging process, which were up- or down-regulated by fold changes, respectively, across all of the group-points (Table 1C). Detailed information on aging related genes is provided in Supplementary data for Table 3.

**Table 1:** Number of differentially expressed genes by aging process.

**(A) Number of DEGs.**					
**No**	**Group 1**	**Group 2**	**Numbers of genes**		
			**Sum**	**Up (G2 only)**	**Down (G1 only)**
1	Y1-4	O1-4	1,275	569 (231)	706 (299)
2	Y1-4	O5-8	1,157	554 (275)	603 (145)
3	Y5-8	O1-4	1,175	722 (261)	453 (122)
4	Y5-8	O5-8	971	551 (257)	420 (32)
5	Y1-4 and Y5-8	O1-4 and O5-8	3,399	1,774 (136)	1,625 (61)
**(B) Number of DEGs mapped into the well annotated genes.**					
**No**	**Group 1**	**Group 2**	**Numbers of genes**		
			**Sum**	**Up (G2 only)**	**Down (G1 only)**
1	Y1-4	O1-4	658	297 (10)	361 (15)
2	Y1-4	O5-8	623	218 (7)	405 (14)
3	Y5-8	O1-4	670	395 (8)	275 (21)
4	Y5-8	O5-8	568	234 (6)	334 (16)
5	Y1-4 and Y5-8	O1-4 and O5-8	2,570	1,279 (3)	1,291 (7)
**© Number of DEGs common between each sample comparison.**				
**Group 1**	**Group 2**	**Numbers of genes**	
		**Sum**	**Up**	**Down**
Young	Old	119	40	79

**Table 2:** Primer.

hCACNA1S	Forward	GACATAATTCCCGCTGCCTG
	Reverse	GTTTCCATTCTTCACCCGCC
hCACNA1A	Forward	ATCGTCTTCACCTCCCTCTTC
	Reverse	GCCCAGAACAGTCACAAAGTC
hCACNA1B	Forward	TTGCCTACTTCTACTTCGTCTCC
	Reverse	TCACAGCCACAAAGAGGTTC
hCACNA1C	Forward	AACAAGGACTGGTGGGAAAG
	Reverse	TGCAAATCACACGATATAGCAC
hCACNA1D	Forward	TTTGACTGCTTCGTCGTGTG
	Reverse	GGTTGCTCAAGGAGTTCCAG
hCACNA1E	Forward	TCCAGTTGGCTTGTATGGAC
	Reverse	AACGTCTCATGGAGCTAGGG
hCACNA1F	Forward	CACCTCCAGTCAGCCCAGT
	Reverse	TCTTGCTTGTTTTGCCCTTT
hCACNA1G	Forward	GGGCATCGAATACCACGA
	Reverse	GCAAAGAGGCTGGTGAAGAC
hCACNA1H	Forward	TCAACGTCATCACCATGTCC
	Reverse	GCCTCGAAGACAAACACGA
hCACNA1I	Forward	GTACTTCAACCGGGGCATC
	Reverse	ATCATCTCCAGGGCAAACA
Col1a1	Forward	ACG AAG ACA TCC CAC CAA TC
	Reverse	ATG GTA CCT GAG GCC GTT C
Col1a2	Forward	GGT CAG CAC CAC CGA TGT C
	Reverse	CAC GCC TGC CCT TCC TT
Col3a	Forward	CCTGGAGCCCCTGGACTAATAG
	Reverse	GCCCATTTGCACCAGGTTCT
Elastin	Forward	TTCTGGGAGCGTTTGGAG
	Reverse	CCTTGAAGCATAGGAGAGACCT
HAS-1	Forward	TGTGACTCGGACACAAGGTTG
	Reverse	GCCT CAAGAAACTGCTGCAA
HAS-2	Forward	ATCCCATGGTTGGAGGTGTT
	Reverse	TGCCTGTCATCACCAAAGCT
MMP-1	Forward	GATGGACCTGGAGGAAATCTTG
	Reverse	TGAGCATCCCCTCCAATACC
MMP-2	Forward	GCACCCATTTACACCTACACCAA
	Reverse	AGAGCTCCTGAATGCCCTTGA
MMP-8	Forward	GACCAACACCTCCGCAAATT
	Reverse	CCCCAAAGAATGGCCAAAT
MMP-9	Forward	GGACGATGCCTGCAACGT
	Reverse	ACAAATACAGCTGGTTCCCAATC
GAPDH	Forward	GGAGTCAACGGATTTGGTCGTA
	Reverse	GGCAACAATATCCACTTTACCAGAGT

### Immunohistochemical stain and Western blot for voltage-gated calcium channels genes

**Figure 1:**
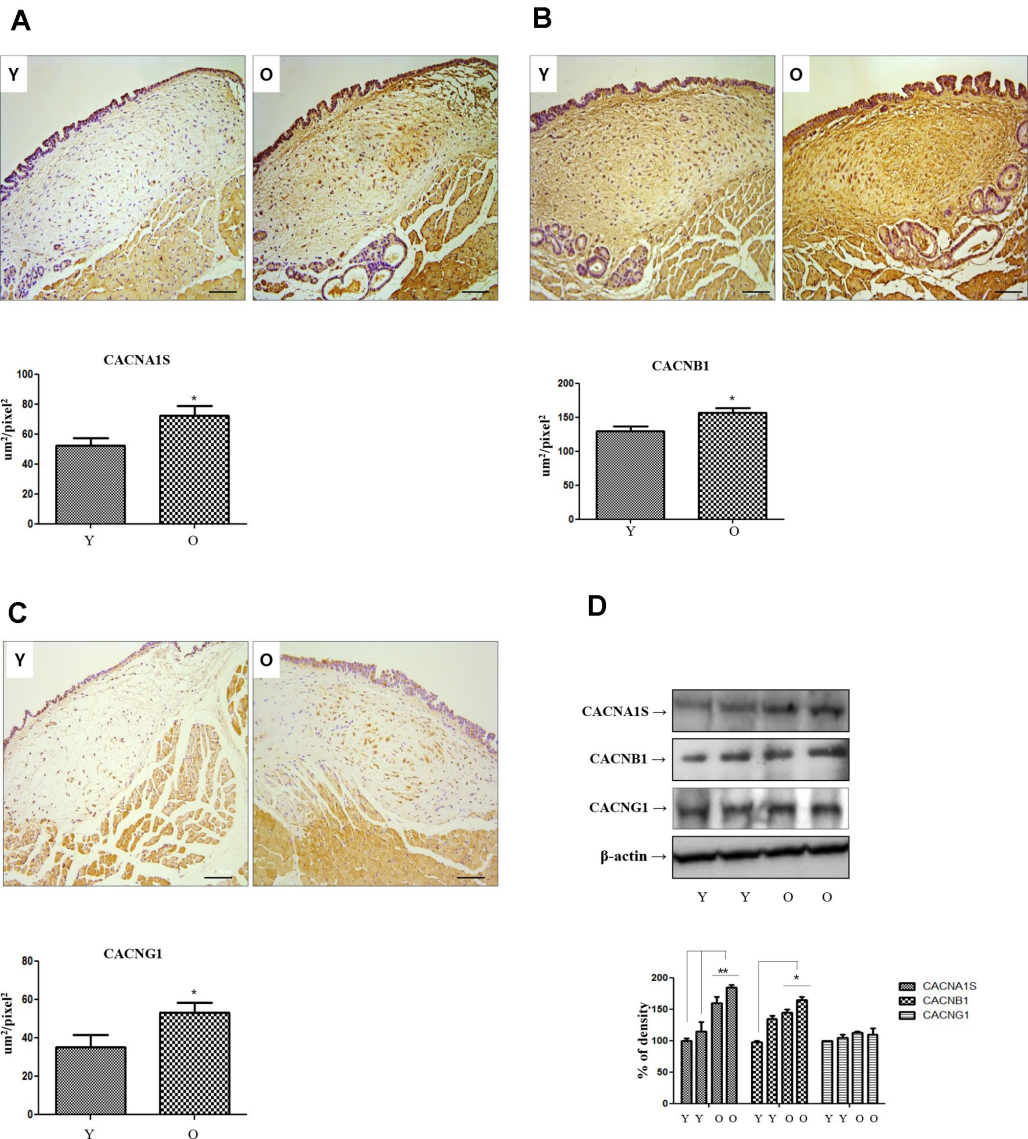
**Immunohistochemistry of CACNA1s, CACNB1 and CACNG1 genes.** Expression of CACNA1S (**A**), CACNB1 (**B**) and CACNG1 (**C**) in IHC staining finding (X200) increase significantly in old compared to young rats. In western blot analysis of these 3 genes, only CACNA1S is increased in primary cultured lamina propria fibroblasts of old compared to young rats (**D**). IHC, Immunohistochemistry. One-way ANOVA test; *p<0.05.

The results of NGS analysis showed that three of the 40 genes that were commonly increased in the lamina propria of the old rats compared to the young rats were calcium channel related genes. So, we selected 3 calcium channel related genes: voltage-gated calcium channels (VGCC) subunit alpha1 S (CACNA1S). VGCC auxiliary subunit beta 1 (CACNB1), and VGCC auxiliary subunit gamma 1(CACNG1). Immunohistochemical staining was performed for CACNA1S, CACNB1 and CACNG1. The expression of all three genes (CACNA1S, CACNB1 and CACNG1) were significantly increased in lamina propria of old rats compared to young rats (Figure 1A–1C). Western blotting was performed on three calcium channel related genes in the primary-culture rat vocal fold fibroblasts of young and aging rats, only CACNA1S was increased compared to young rats (Figure 1D).

### Knockdown of voltage-gated calcium channels genes reduces collagen synthesis on hVFFs

**Figure 2:**
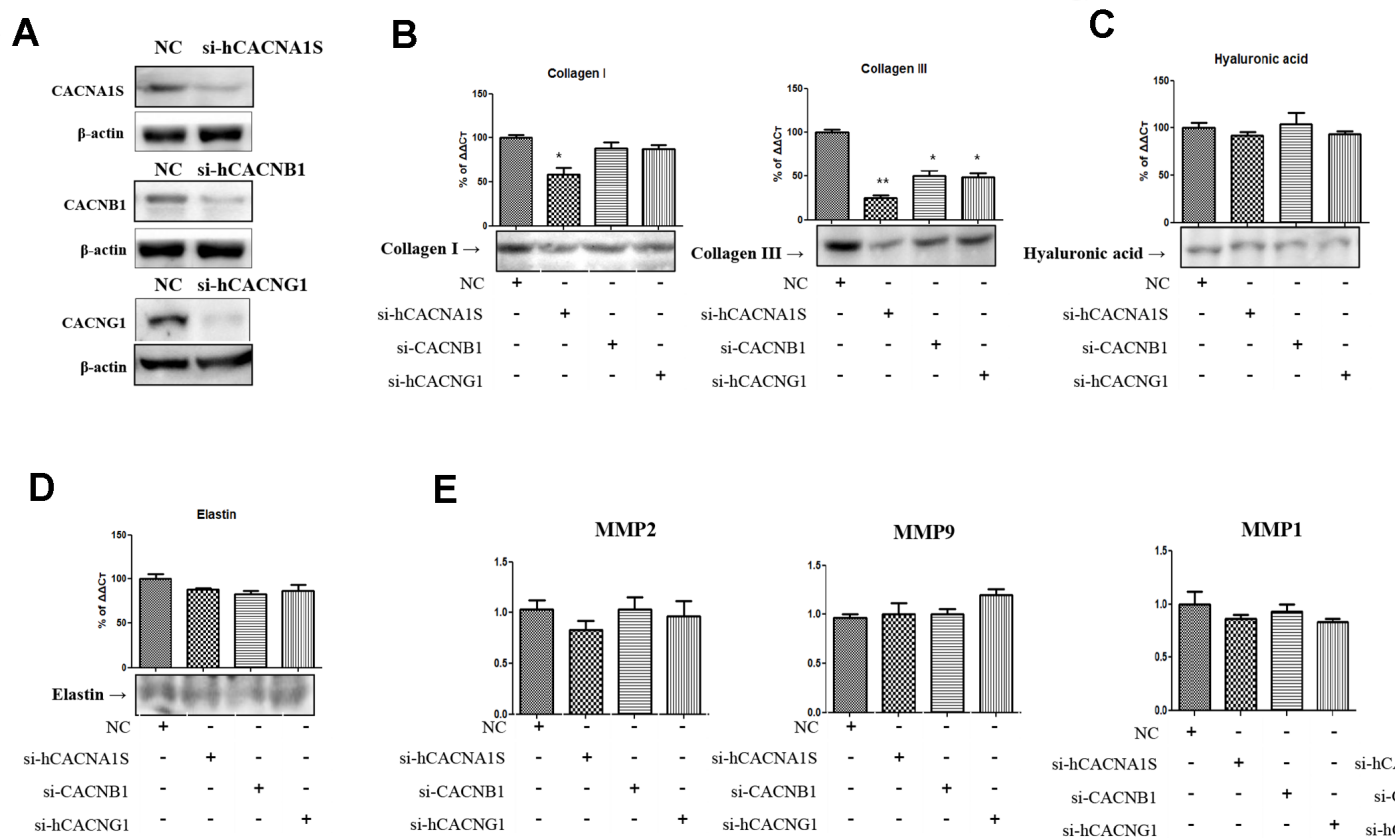
**Alteration of extracellular matrix by knockdown of CACNA1s, CACNB1 and CACNG1 genes in hVFFs.** CACNA1s, CACNB1 and CACNG1 genes are effectively knocked down (**A**). CACNA1S knockdown cells significantly inhibit the synthesis of collagen I and III protein compared to CACNB1 and CACNG1 knock down cells (**B**). However, the synthesis of hyaluronic acid and elastin were not affected by knockdown of CACNA1s, CACNB1 and CACNG1 genes (**C** and **D**). These knockdown cells did not show a significant change in the expression of MMP-1, 2, 8, and 9 (**E**). Relative gene expression (fold change) was normalized to the respective housekeeping gene (18s RNA) controls. MMPs, matrix metalloproteinases. One-way ANOVA test; NS as no significant, *p<0.05, and **p<0.01.

The three calcium channel genes (CACNA1S, CACNB1 and CACNG1) which were significantly increased by the immunohistochemistry and NGS studies in the aging lamina propria of rat were expressed as hVFFs (Figure 2A). To investigate the relationship between these genes and the alteration of extracellular matrix in aging lamina propria, the changes of collagen and hyaluronic acid were observed by knockdown using siRNA for these calcium channel genes. We confirmed by qPCR and western blotting under non-reducing condition whether these genes were effectively knocked down (Figure 2A). After that, the protein expression of collagen I, III, hyaluronic acid and elastin was examined. The results show that these knockdown cells suppressed collagen I and III expression. Especially, CACNA1S knockdown cells significantly inhibited collagen I and III protein synthesis compared to CACNB1 and CACNG1 knock down cells (Figure 2B). However, hyaluronic acid and elastin were not affected by knockdown of these calcium channels genes (Figure 2C and 2D). These knockdown cells did not show a significant change in the expression of MMP-1, 2, 8, and 9 associated with collagen degradation (Figure 2E).

### Proliferation and viability of hVFFs after verapamil treatment

**Figure 3:**
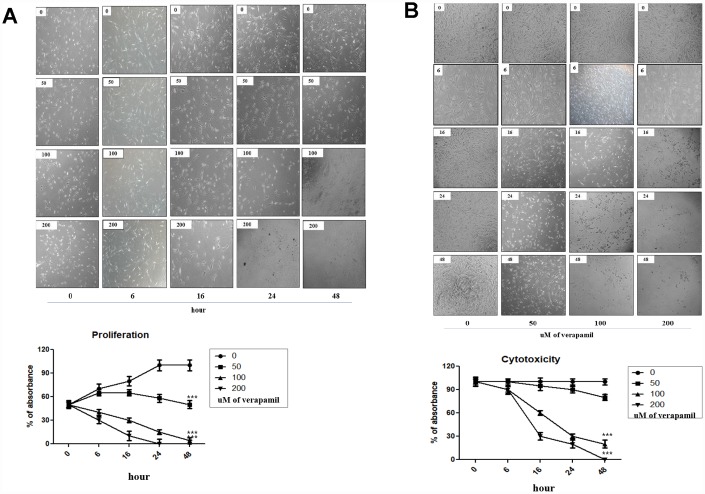
**Cellular proliferation and viability by verapamil in hVFFs.** Photographs and graphs show the changes of the proliferation (**A**) and viability (**B**) of hVFFs at various time points depending on the concentration of verapamil. Verapamil treatment reduced the proliferation and viability of hVFFs cells as dose- and time-dependent manner. Represented are light microscopic images of hVFFs for general morphology. One-way ANOVA test; ***p<0.001.

The effect of verapamil, one of the most commonly used VGCC blockers in clinical practice, on the proliferation and viability in hVFFs was investigated. MTT assay results showed that verapamil treatment reduced the proliferation and viability of hVFFs cells dose- and time-dependent manner (Figure 3A and 3B). As the concentration of verapamil increased, the proliferation and viability of hVFFs decreased. Also, the proliferation and viability of hVFFs decreased with increasing treatment time of verapamil.

### Expression of human CACNA (hCACNA1) subtypes in hVFFs cell lines

**Figure 4:**
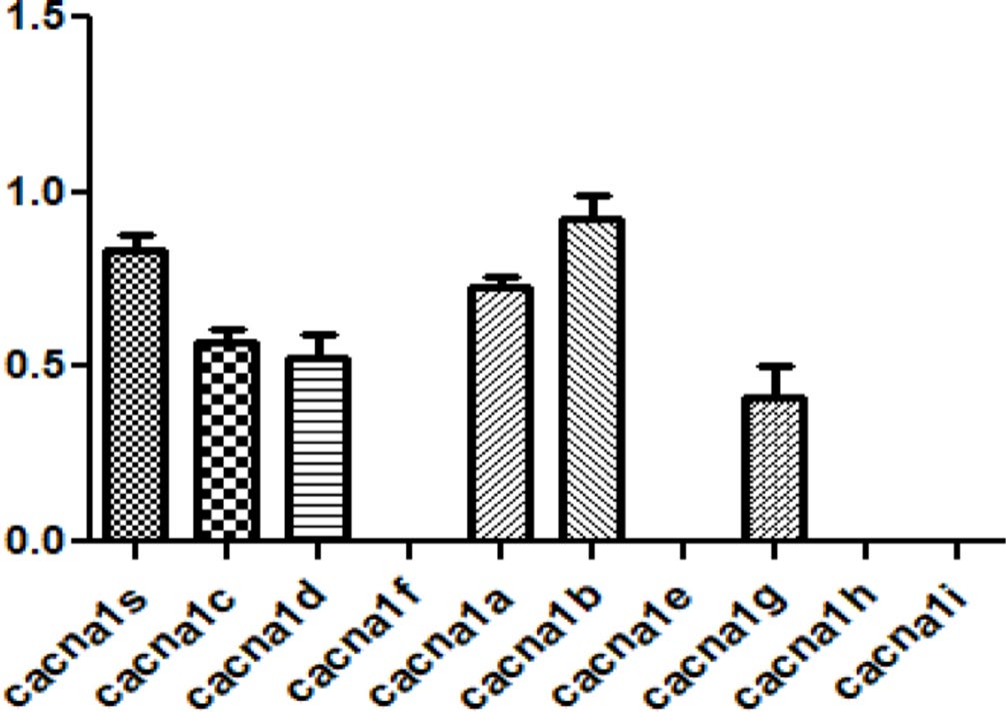
**Expression of voltage-gated calcium channel subunit alpha subtypes in hVFFs.** The expression of 10 hCACNA1 subtypes are investigated in hVFFs. hCACNA1B, hCACNA1S, and hCACNA1A are highly expressed compared to other subtypes.

CACNA1S that is most relevant to changes in aging-related ECM is one of the subtypes of voltage-gated calcium channels subunit alpha1 (CACNA1). CACNA1 is the primary function unit of the calcium channel and has 10 sub-types [15]. So, the expression of other hCACNA1 subtypes was investigated in hVFFs. hCACNA1B, hCACNA1S, and hCACNA1A are highly expressed compared to other subtypes (Figure 4).

### Alteration of CACNA1s gene and ECM protein with verapamil treatment in hVFFs

**Figure 5:**
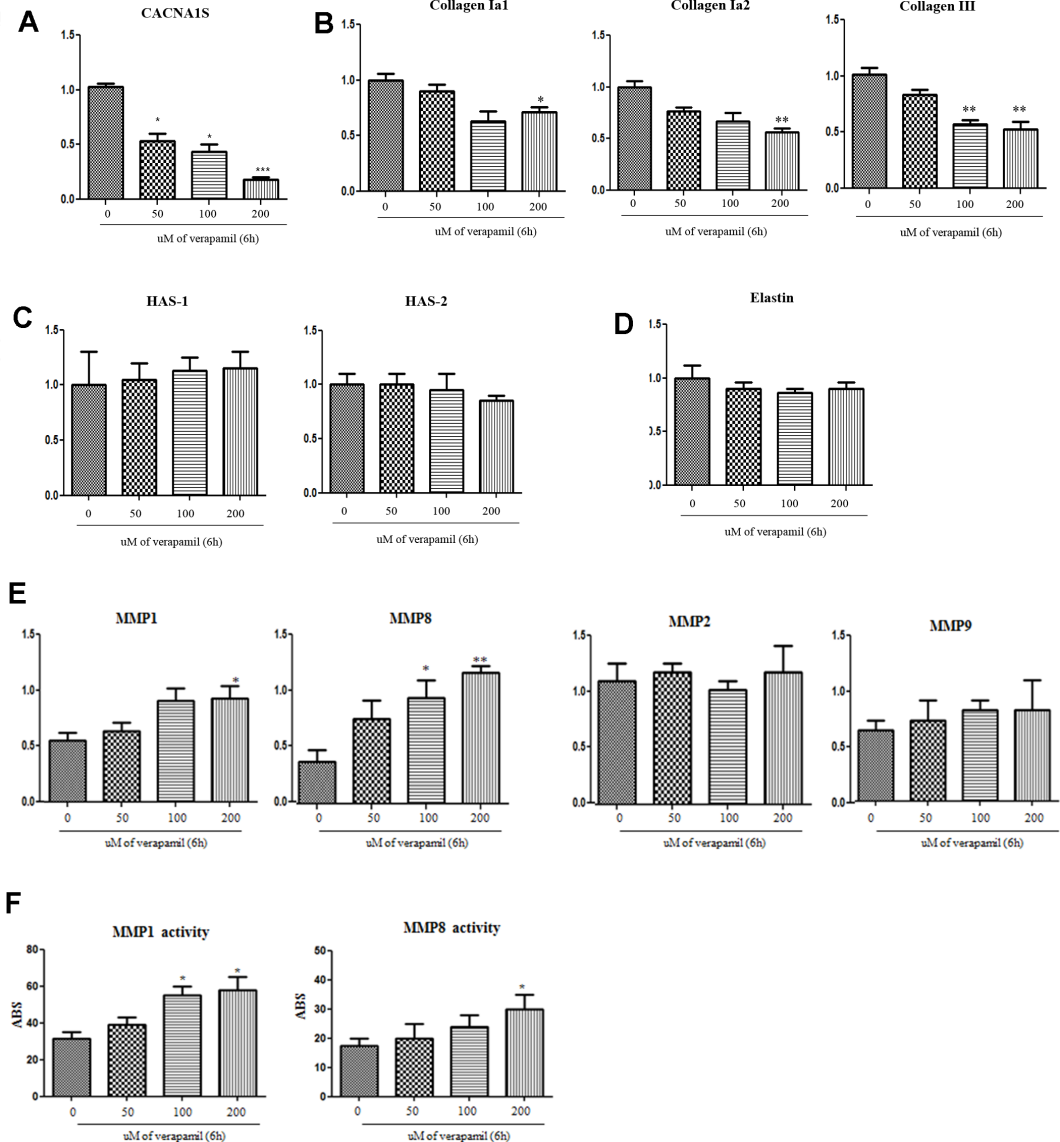
**Alteration of CACNA1s gene and ECM protein with verapamil treatment in hVFFs.** After the verapamil treatment for 6 h in hVFFs, the expression of CACNA1S decreases in RT-PCR as dose-dependent manner (**A**). The synthesis of collagen I alpha 1 and 2 and collagen III in hVFFs decrease according to the concentration of verapamil (**B**). However, there are no effect on the synthesis of HA or elastin (**C** and **D**). MMP-1 and -8 are increased the expression and activity as the concentration increased (**E** and **F**). However, there was no change in the expression of MMP-2 and -9 (**E**). Relative gene expression (fold change) was normalized to the respective housekeeping gene (18s RNA) controls. HAS, hyaluronic acid synthase. MMPs, matrix metalloproteinases. One-way ANOVA test; NS as no significant, *p<0.05, **p<0.01 and ***p<0.001.

When the verapamil was treated with hVFFs for 6 hours, the expression of CACNA1S decreased in qPCR and Western blot (Figure 5A). Also, the synthesis of collagen I and III in hVFFs decreased according to the concentration of verapamil (Figure 5B). However, there was no effect on the synthesis of hyaluronic acid or elastin (Figure 5C and 5D). MMP-1 and -8, which are associated with the degradation of collagen, increased the expression and activity as the concentration increased (Figure 5E and 5F). However, there was no change in the expression of MMP-2 and -9 (Figure 5E).

## DISCUSSION

The normal aging process is associated with increased amounts of oxidative stress and perturbed cellular energy metabolism involving impaired mitochondrial function [16]. It is also known that the oxidative stress and impaired energy metabolism are involved in the development of various diseases such as hypertension, diabetes, cardiovascular dysfunction, and Alzheimer’s disease as well as aging [17]. Reactive oxygen species (ROS) is one of the major players in cellular growth, senescence, aging and death [18]. Dysregulation or dyshomeostasis of calcium is associated with the regulation of rhythmicity and contractility in the heart and the pathogenesis of aging-related neurodegeneration in brain [19]. Cellular calcium signaling pathways are regulated by other cellular signaling systems such as ROS [20]. In aging hippocampal neurons, L-type calcium channels (CACN) are increased, and this increase is related to age-dependent cognitive decline [21, 22]. The increase in L-type CACN activity appears to be a primary mechanism for triggering calcium dyshomeostasis in brain aging [23]. Cellular calcium overload is believed to play a key role in the death of neurons by a stroke [24]. T-type calcium channels inhibitors drastically reduce the ischemic-induced delayed cell damage [25]. Verapamil inhibits calcium influx and prevents neural damage and memory impairment caused by severe hypoglycemia in diabetes mellitus [26]. The calcium channel is one of important molecules involved in the mechanism of aging and the mechanism of various diseases.

According to literature review, there has been no study on the expression or role of VGCCs in the lamina propria of the vocal fold. This study was the first to confirm that the VGCCs are expressed in the lamina propria of vocal fold through NGS and immunohistochemical stain. We also found that the CACNA1S, CACNB1, and CACNG1 were significantly increased in the lamina propria of aged rats in immunohistochemical staining. When si-hCACNA1S was treated with hVFFs, the synthesis of collagen I and III was decreased, but HA and elastin were not changed. There was no significant change in the synthesis of collagen I, HA and elastin when treated with si-hCACNB1 and si-hCACNG1 in hVFFs. These suggest that the increase of CACNA1S in the aging lamina propria may be associated with an increase in collagen I and III, one of the important findings of the aging vocal cords. Further studies on the role and function of increased these genes in the lamina propria of aging vocal ford are needed. So, a better understanding of the cellular and molecular mechanism for cellular calcium homeostasis during aging my lead to novel approaches for therapeutic target.

The VGCCs control many critical physiological process, including hormone and neurotransmitter release, cell migration, gene transcription, and muscle contraction [27, 28]. VGCCs consist of an assembly of subunits that together form a functional channel. The main calcium channel α1 (CACNA1) subunit associates with auxiliary subunit β, α2-δ and sometimes γ to form the VGCC complex. Although CACNA1 alone is able to conduct current, the auxiliary subunits allow for proper activation and inactivation properties [30]. Ten different genes for CACNA1 have been identified [31]. There are differences in subtypes that are mainly expressed in organ or tissue. CACNA1S subtype is predominately found in the skeletal muscle and CACNA1C is located in cardiac tissue and brain. CACNA1D is generally in the central nervous system and auditory hair cells [32, 33]. According to literature review, there is no study on the expression patterns of CACN subtypes in human vocal fold fibroblasts. In our study, hCACNA1B, hCACNA1S and hCACNA1A were expressed higher than other subtypes in human VFFs. Further studies on the role and function of expressed these genes in the lamina propria of vocal fold are needed.

Fibroblasts are the most abundant cell type in the lamina propria and play a major role in synthesizing components of ECM and the regulation of collagen degradation. The important ECM-related changes in the aging-related lamina propria are the increased deposition of collagen I and III, the decreased deposition of HA and elastin, and the decreased activity of matrix metalloproteinases (MMP)-2 and 9 [6–9]. Although the MMP-2 and -9 increases in lamina propria of aging vocal fold, there is no report about the expression of MMP-1 and 8 in aging vocal folds [9]. MMP-2 (gelatinase A) has type IV collagenolytic activity and MMP-9 (gelatinase B) has type V collagenolytic activity. These two important enzymes are known to be important for aging vocal folds and tissue remodeling of wound healing, but they are not involved in the collagen degradation activity of increased collagen I and III, which is an important characteristic of the ECM of the aging vocal folds [34, 35]. MMP-1 and -8 are important enzymes involved in collagen I and III degradation. It is important that the novel therapeutic target for the alterations of aging-related ECM in lamina propria reduces the synthesis of collagen I and III, which are important features of ECM, and increases the degradation of collagen I and III.

There is no study about the relationship between the CACN expression and ECM changes in vocal fold fibroblasts. However, there are some studies for the ECM remodeling by calcium channel blockers (CCBs) in the dermal fibroblast or cardiac fibroblast [36–38]. Yue H et al. reported the decrease in MMP-2 during treatment with amlodipine, one of CCBs, in cardiac fibroblasts, but neither verapamil nor diltiazem altered MMP-2 expression [36]. However, all CCBs, including verapamil, reduced the deposition of collagen type I in the ECM of dermal fibroblasts [37].

Verapamil is a first generation calcium channel blocker widely used for treatment of hypertension through inhibits the transmembrane influx of extracellular calcium ions into myocardial and vascular smooth muscle cells, causing dilatation of the main coronary and systemic arteries and decreasing myocardial contractility. We chose verapamil to focus on the previous report that it affects collagen synthesis as well as the calcium channel blocking effect of verapamil. Verapamil inhibits scar formation by inhibiting fibroblast adhesion and proliferation *in vitro* and diminished the secretion of extracellular matrix from keloid and hypertrophic scar *in vivo*, suppressed type I and III collagen secretion [38, 39].

The alterations of verapamil-treated fibroblast are represented by morphological changes, low cytosolic calcium concentration, discrete reorganization of actin cytoskeleton, and increase of MMP-1 production and activity [40]. In the our study, when verapamil was treated in the hVFFs, the expression of hCACNA1S and the synthesis of collagen I and III was significantly decreased, and the expression and activity of MMP-1 and MMP-8 were increased. Verapamil not only inhibits the production of collagen I and III but also increases collagen I and III degradation by increasing the activity of MMP-1 and -8 in hVFFs. These results may be due to the decrease of intracellular calcium influx by calcium channel dysfunction, which is caused by the decrease of CACNA1S, but it is necessary to study the exact mechanism of CCBs effect to modify ECM.

**Figure 6:**
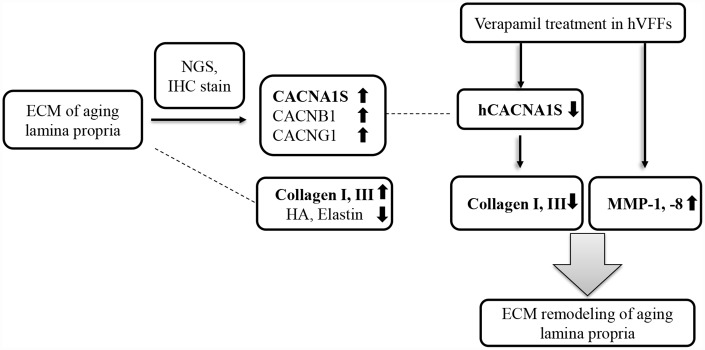
**Summary of our study.** CACNA1S, CACNB1, and CACNG1 are significantly increased in the NGS study and immunohistochemistry in the lamina propria of aging vocal folds. The synthesis of collagen I and III of hVFFs with si-CACNA1S are reduced significantly. When verapamil is treated in hVFFs, the expression of CACNA1S and the synthesis of collagen I and III are decreased and the expression of MMP-1 and 8 are increased. Voltage gaited calcium channel, especially CACNA1S, has promising potential as a novel therapeutic target for remodeling ECM of lamina propria.

There are several studies to reduce the scar formation using the effect of ECM remodeling of the CCBs. CCBs have been reported to inhibit peripheral nerve scars by reduced the axon resistance [41]. Intralesional verapamil reduced the keloid and hypertrophic scars [42, 43]. Also, verapamil is capable of inhibiting the production of cytokines, cellular proliferation, and the biosynthesis of the ECM [44]. The anti-scar effect of verapamil is thought to be associated with the collagenase activity of the ECM, the synthesis and secretion of collagen and fibronectin, and the alteration of the metabolism and proliferation of fibroblast. Our results suggest that the verapamil intracordal injection and knockdown of CACNA1S are likely to be a novel therapeutic modality that regulates the ECM of the vocal fold lamina propria associated with scar or aging (Figure 6). In conclusion, CACNA1S, CACNB1, and CACNG1 were significantly increased in the NGS study and immunohistochemistry in the lamina propria of aging vocal folds. The synthesis of collagen I and III of hVFFs with si-CACNA1S was reduced significantly. When verapamil was treated in hVFFs, the expression of CACNA1S and the synthesis of collagen I and III were decreased and the expression of MMP-1 and 8 were increased. These results suggest that some calcium channels may be related with the alteration of aging-related ECM in vocal folds. Voltage gaited calcium channel, especially CACNA1S, has promising potential as a novel therapeutic target for remodeling ECM of aging lamina propria.

## MATERIALS AND METHODS

### Animal

The animal protocol used in this study was reviewed and approved beforehand by the Pusan National University-Institutional Animal Care and Use Committee (PNU-IACUC) with respect to ethicality and scientific care. To investigate the difference of genes expression of age-related lamina propria in rat vocal fold during aging process, we used 6 and 22 months old male Sprague-Dawley rats (n=8, each group) for NGS. Six and 22 months old male SD rats (n=12, each group) were used for immunohistochemistry validation and western blotting of the molecules proposed in the NGS study.

### Tissue preparation for NGS and RNA QC, library construction, and sequencing

Larynges were harvested immediately after sacrifice and frozen with liquid nitrogen and storage in -80°C freezer for RNA-seq and real time qPCR. NGS was performed on 2 young samples (young 1-4, young 5-8) and 2 old samples (old 1-4, old 5-8). Tissue RNA extraction was commissioned by Teragene (Osong, Korea). Ahead of cDNA library construction, the 2ug of total RNA and magnetic beads with Oligo (dT) were used to enrich poly (A) mRNA from it. Then, the purified mRNAs were disrupted into short fragments, and the double-stranded cDNAs were immediately synthesized. The cDNAs was subjected to end-repair, poly (A) addition, and connected with sequencing adapters using the TruSeq RNA sample prep Kit (Illumina, CA). The suitable fragments automatically purified by BluePippin 2% agarose gel cassette (Sage Science, MA) were selected as templates for PCR amplification. The final library sizes and qualities were evaluated electrophoretically with an Agilent High Sensitivity DNA kit (Agilent Technologies, CA) and the fragment was found to be between 350–450 bp. Subsequently, the library was sequenced using an Illumina HiSeq2500 sequencer (Illumina, CA).

### Transcriptome data analysis

Low quality reads were filtered according to the following criteria; reads contain more than 10% of skipped bases (marked as ‘N’s), reads contain more than 40% of bases whose quality scores are less than 20 and reads whose average quality scores of each read is less than 20. The whole filtering process was performed using the in-house scripts. Filtered reads were mapped to the human reference genome, Ensembl release 72, using the aligner STAR v.2.3.0e [45, 46]. Gene expression level was measured with Cufflinks v2.1.1 using the gene annotation database of Ensembl release 72. Non-coding gene region was removed with mask option. To improve the accuracy of measurement, multi-read-correction and fragbias-correct options were applied. All other options were set to default values.

### DEGs (Differentially Expressed Genes) analysis

For differential expression analysis, gene level count data were generated using HTSeq-count v0.5.4p3 tool with the option “-m intersection-nonempty” and -r option considering paired-end sequence. Based on the calculated read count data, DEG were identified using the R package called TCC. TCC package applies robust normalization strategies to compare tag count data. Normalization factors were calculated using the iterative DEGES/edgeR method. Q-value was calculated based on the p-value using the p.adjust function of R package with default parameter settings. Differentially expressed genes were identified based on the q-value threshold less than 0.05.

### Laryngeal preparation and immunohistochemistry

For immunohistochemical stain, primary anti-CACNA1S, anti-CACNB1 (Abcam, Cambridge, United Kingdom) and anti-CACNG1 (Life Technologies, Rockville, MD) were used. The following goat-anti rabbit secondary antibodies were used for double-staining with DAB staining. We selected proper central part of vocal fold tissue for representative figures using undertaken at 200X pictures by light microscope (Leica DM4000/600M, Versatile upright microscope for materials analysis).

### Rat lamina propria fibroblast isolation and Western blot

We isolated whole lamina propria of vocal fold with a needle of syringe under microscopic view for the primary culture of rat vocal fold fibroblast. Lamina propria tissues were dissociated and digested with 0.1% trypsin and 200 unit/ml type I collagenase (Gibco, Carlsbad, CA) at 37 °C for 1 hour, strained through a 40-μm filter, rinsed, resuspended in Dulbecco's modified Eagle's medium (DMEM)/F12 medium containing 10% fetal bovine serum (FBS) and 100 U/mL penicillin-streptomycin (Sigma-Aldrich, St. Louis, MO), and plated on culture plates. The dishes were kept at 37°C in a cell culture incubator with 5% CO_2_-95% air. The medium was changed every 2 to 3 days until cells had migrated from the explant. The remaining tissue pieces were removed, and cells were allowed to reach subconfluence (ie, 90% confluent). For Western blotting analysis, cells were harvested and lysed in PROPREP lysis buffer (Invitrogen). Equal amounts of protein were separated on 10-12% SDS-PAGE gels (Life Technologies) as described by Laemmli (Laemmli, 1970) with primary anti-CACNA1S, anti-CACNB1 (Abcam, Cambridge, United Kingdom) and anti-CACNG1 (Life Technologies, Rockville, MD) were used.

### Gene knockdown with siRNA with human vocal fold fibroblast cell lines (hVFFs)

The hVFFs were kindly provided by Professor Susan Thibeault of the University of Wisconsin, USA [47]. The small interfering RNA (siRNA) oligonucleotides that specifically target human CANCA1S, CACNB1 and CACNG1 were prepared and synthesized by Dharmacon Inc. (Austin, TX, USA). A negative control siRNA (scrambled) was included to monitor nonspecific effects. Cells were transfected with 60 pmoles of ON-TARGETplus human CANCA1S, CACNB1 and CACNG1 siRNA against calcium channel genes for 24~36h using the Lipofectamin 2000 transfection reagent (invitrogen) in OPTIMEM medium (Invitrogen) according to the manufacturer’s protocol. Scramble siRNA using as negative control. The efficiency of knockdown is confirmed with Western blot analysis under non-reducing and reducing condition.

### Verapamil treatment and real time-PCR for calcium channel related genes and ECM related genes

For all experiments, cells were treated with various dosage of verapamil. After time period, cells were incubated with MTT at 37 °C for 4 h, and the precipitate was dissolved in DMSO. Subsequently, the absorbance (optical density, OD) at 570 nm was measured using a microplate reader (Model 680; Bio-Rad Laboratories, Hercules, CA, USA) and cell viability was calculated according to the % formula. Cellular RNA was extracted using the TRIzol system (Life Technologies, Rockville, MD). Reverse Transcription Kit (Applied Biosystems, Foster City. California) was used to perform reverse transcription according to the manufacturer's recommended reaction protocol. Real-time PCR was performed according to the SYBR Green PCR protocol (Applied Biosystems Foster City CA). Gene-specific PCR products were continuously measured by an ABI PRISM 7900 HT Sequence Detection System (PE Applied Biosystem Norwalk, CT). The information on primer sequences are as following Table 2.

### Matrix Metalloproteinase (MMP) activity assay

The general activity of MMP enzyme was determined using an assay kit purchased from Abcam (Cat No. ab112146) according to the manufacturer’s protocol. Cells were treated with vehicle (control) and various dosage of verapamil and the MMP activity was assayed in the conditioned media. A kinetic measurement was then performed for the MMP activity by using a microplate reader with a filter set of Ex/Em = 490/ 525 nm.

### Statistical analysis

Unless otherwise noted, all quantitative data were reported as the mean standard error of the mean from at least three parallel repeats. Two-way analysis of variance was used to determine significant differences between groups in which P < 0.05 was considered statistically significant. The analyses were performed using Graph Pad Prism 5 (Graph Pad software, La Jolla, CA, USA).

## Supplementary Material

Supplementary data for Table 1

Supplementary data for Table 2

Supplementary data for Table 3
